# Phytonutrients in the promotion of healthspan: a new perspective

**DOI:** 10.3389/fnut.2024.1409339

**Published:** 2024-07-12

**Authors:** Emma F. Jacquier, Amira Kassis, Diana Marcu, Nikhat Contractor, Jina Hong, Chun Hu, Marissa Kuehn, Christopher Lenderink, Arun Rajgopal

**Affiliations:** ^1^Neat Science, Chatel-Saint-Denis, Switzerland; ^2^School of Molecular Biosciences, College of Medical, Veterinary and Life Sciences, University of Glasgow, Glasgow, United Kingdom; ^3^Amway Innovation and Science, Ada, MI, United States

**Keywords:** aging, nutrition, healthspan, phytonutrients, vitality, intrinsic capacity

## Abstract

Considering a growing, aging population, the need for interventions to improve the healthspan in aging are tantamount. Diet and nutrition are important determinants of the aging trajectory. Plant-based diets that provide bioactive phytonutrients may contribute to offsetting hallmarks of aging and reducing the risk of chronic disease. Researchers now advocate moving toward a positive model of aging which focuses on the preservation of functional abilities, rather than an emphasis on the absence of disease. This narrative review discusses the modulatory effect of nutrition on aging, with an emphasis on promising phytonutrients, and their potential to influence cellular, organ and functional parameters in aging. The literature is discussed against the backdrop of a recent conceptual framework which describes vitality, intrinsic capacity and expressed capacities in aging. This aims to better elucidate the role of phytonutrients on vitality and intrinsic capacity in aging adults. Such a review contributes to this new scientific perspective—namely—how nutrition might help to preserve functional abilities in aging, rather than purely offsetting the risk of chronic disease.

## 1 Introduction

By the year 2100, it is projected that a quarter of the world’s population will be aged over 64 (1). Living longer has not necessarily been accompanied by continued good health, therefore while lifespan may have increased, the healthspan has been improving at a much slower pace (2). Healthspan, or healthy aging, is defined as the number of years lived in good physical, cognitive, and emotional health (3).

Diet and lifestyle stand out as pivotal factors in this quest, with nutrition playing a crucial role in modulating multiple cellular aging processes (4–10). Good quality plant-based diets which provide essential nutrients and bioactive phytonutrients, have the potential to impact multiple aging mechanisms, such as, inflammation, metabolism, and cellular repair (10–15). Phytonutrients have been defined as, “*Compounds present in and/or derived from plants that confer a health benefit (including metabolites post consumption)”* (16). These compounds are found in edible plants and have a variety of important functions including protecting plants from various environmental stressors (17). Phytonutrients should ideally be provided via healthy dietary patterns which emphasize plant foods, or they may also be provided in safe, concentrated forms via plant nutraceuticals. Nutraceuticals can be defined as “*A compound or mixture of compounds present in food or food supplements intended to exert a therapeutic effect*” (11, 16).

Recently, the preservation of vitality and intrinsic capacity, as opposed to specifically focusing on the presence and absence of disease, has come to the fore in healthy aging research (7). In this narrative review, we present and discuss the scientific evidence around certain dietary phytonutrients and their modulatory effects on aging at the cellular, organ and functional levels. With more than 10,000 potential phytonutrients, this review has selected phytonutrients with varying levels of research ranging from the emerging to the well-studied. We will discuss this broad range of phytonutrients against the backdrop of the conceptual framework on vitality and intrinsic capacity in aging originally proposed by Beard et al. (18) and recently elaborated by a WHO working group (7). In the context of nutrition, the consideration of this framework in a narrative review helps to contextualize and elucidate how certain phytonutrients might influence biological aging across the aforementioned levels. Furthermore, we identify research gaps and future opportunities for phytonutrients to help influence the vitality and intrinsic capacity of aging adults.

### 1.1 Aging is a multifactorial and multiorgan loss of functional ability

Aging is a multifactorial process, driven by a complex interplay of numerous molecular mechanisms that collectively contribute to the gradual decline in physiological function and increased susceptibility to age-related diseases (19). Understanding the intricacies of aging involves examining both organ or system specific changes and the broader network of interactions among organ systems. For instance, the aging immune system is characterized by immunosenescence, inflammaging, and age-related dysbiosis (20). This progressively leads to a diminishing ability of the body to protect itself, making older adults more susceptible to infections, and less responsive to vaccination (21). In the cardiovascular system, vascular aging results in endothelial dysfunction and impaired vasodilation, placing older adults at a higher risk of cardiovascular disease (CVD). In addition, metabolic dysfunction arising from disrupted nutrient sensing, coupled with low-grade inflammation and oxidative stress, contribute to worsening atherosclerotic plaque, arterial thickness and stiffness further increasing that risk (22). Similar declines are observed in the nervous and musculoskeletal systems with cognitive impairment and sarcopenia as known consequences (23–25).

Aging organs are interconnected, with the biological aging rate in one organ system affecting the aging trajectory of others (26, 27). Consequently, accelerated biological aging in the kidney or the brain, can exert systemic effects on other organ systems through shared pathways, including inflammation, oxidative stress, and hormonal dysregulation (28). This interorgan communication forms a complex network of interactions, leading to the concept of multiorgan aging and therefore comorbidity (29).

### 1.2 Nutrition as a contributor to healthy aging

**Figure 1:**
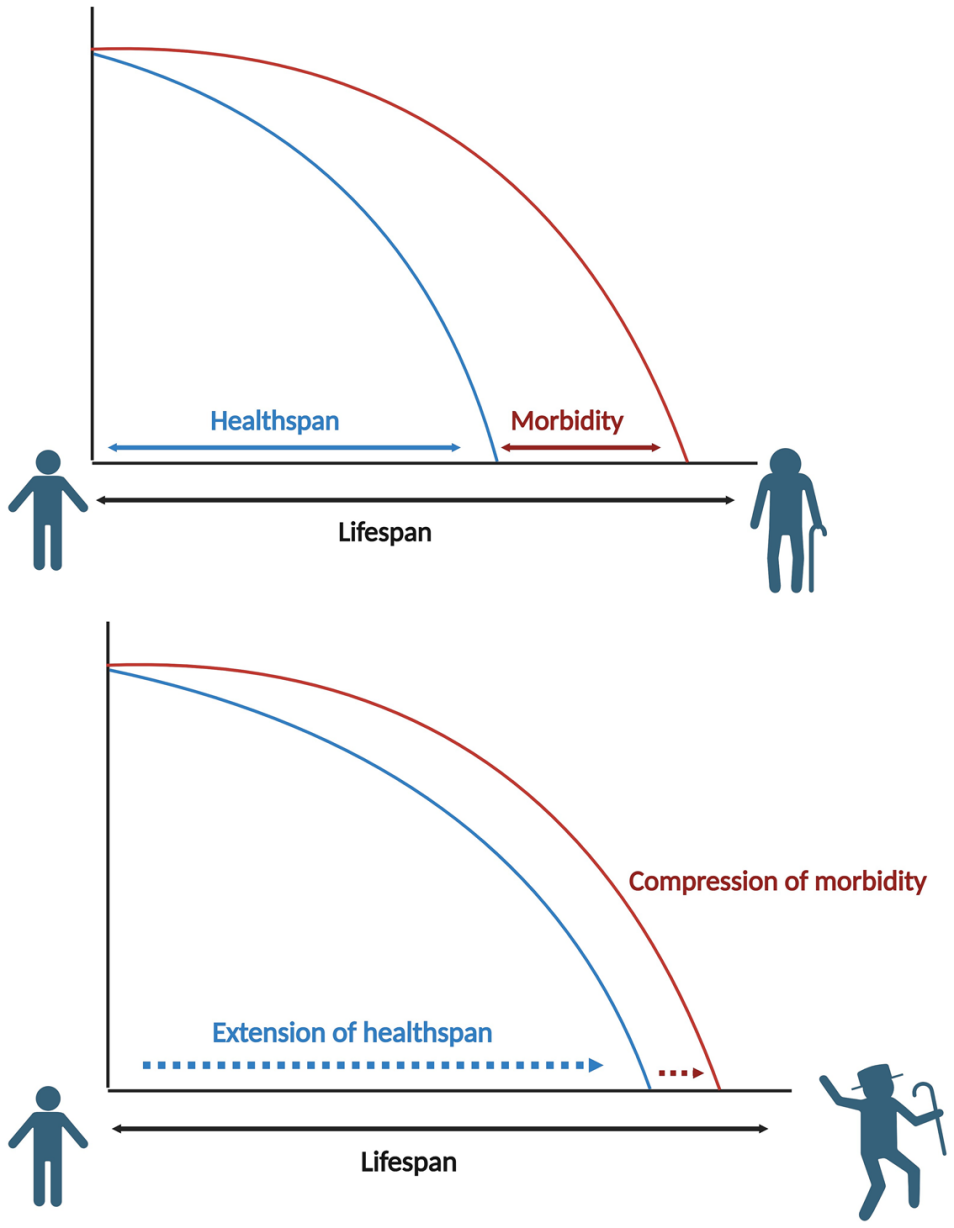
Comparative diagram of lifespan vs. healthspan.

The ideal scenario of healthy aging is that lifespan extension is accompanied by a proportional increase in healthspan, resulting in an older population that lives longer, in better health, with a condensed period of morbidity (Figure 1).

**Figure 2:**
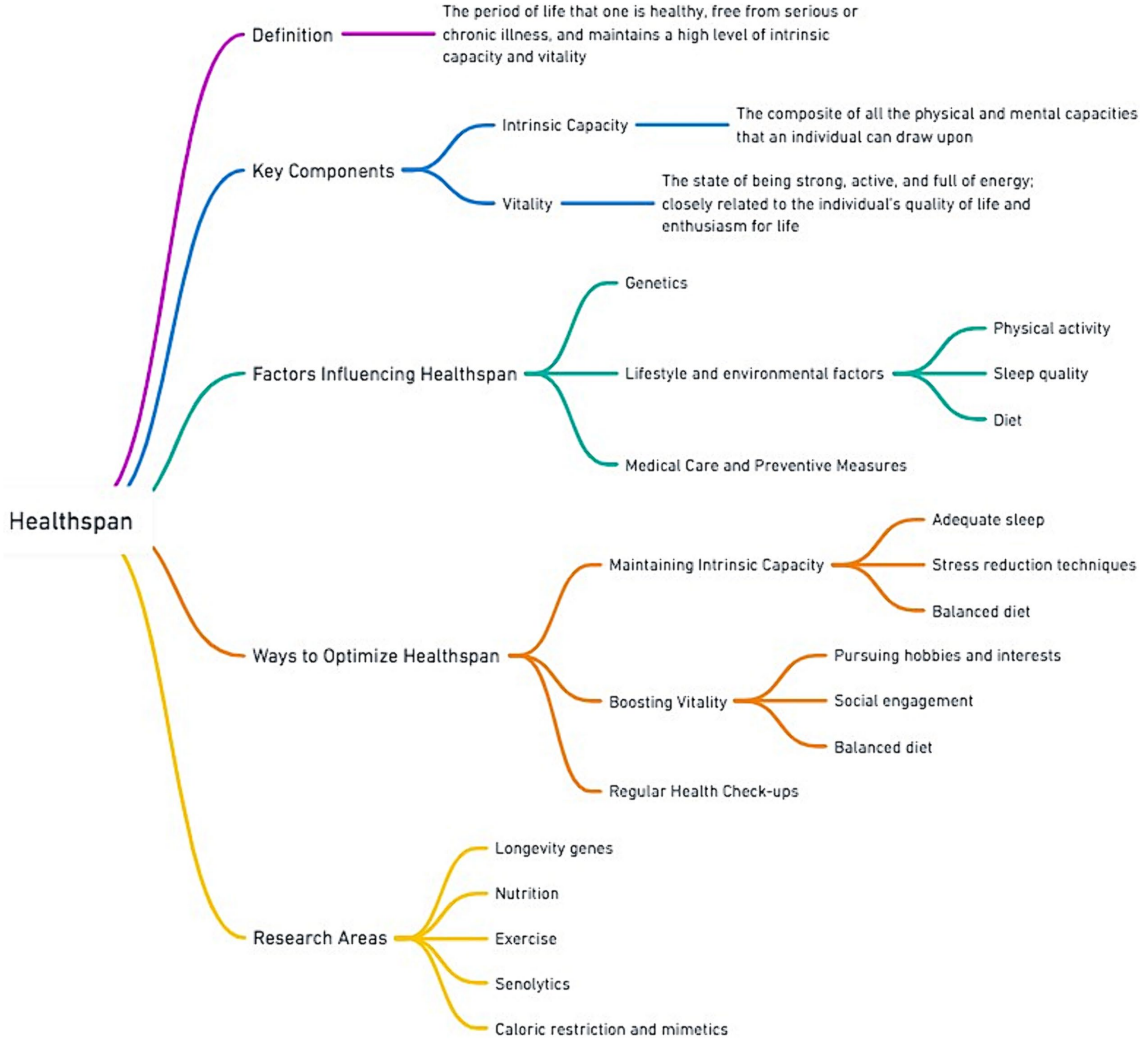
Overview of healthspan with focus on determinants and optimization strategies.

Building on this foundation, Figure 2 provides a *comprehensive* overview of healthspan, detailing its definition, key components, influencing factors, and methods for optimization. Importantly, Figure 2 aims to highlight that the notion of healthspan should recognize that aging is not solely defined by the *absence of disease or compression of morbidity*, but also encompasses the preservation of intrinsic capacity and vitality (30).

In addition, Figure 2 highlights the notion that diet is central to influencing healthspan (14, 22, 31), and interlinked with other lifestyle and environmental factors, such as physical activity, sleep quality, access to medical care and preventive measures. Adequate nutrition is associated with maintaining intrinsic capacity by supporting bodily functions and cognitive health, and it may also play a vital role in boosting vitality (6, 7, 9). Diet and nutrition are also at the heart of current research areas, including studies on longevity genes, the biological impacts of nutrition and exercise synergies, the effects of senolytics, and the potential benefits of caloric restriction and its mimetics (32, 33). Such research underscores the importance of nutritional intervention strategies aimed at promoting healthy aging and enhancing quality of life.

**Figure 3:**
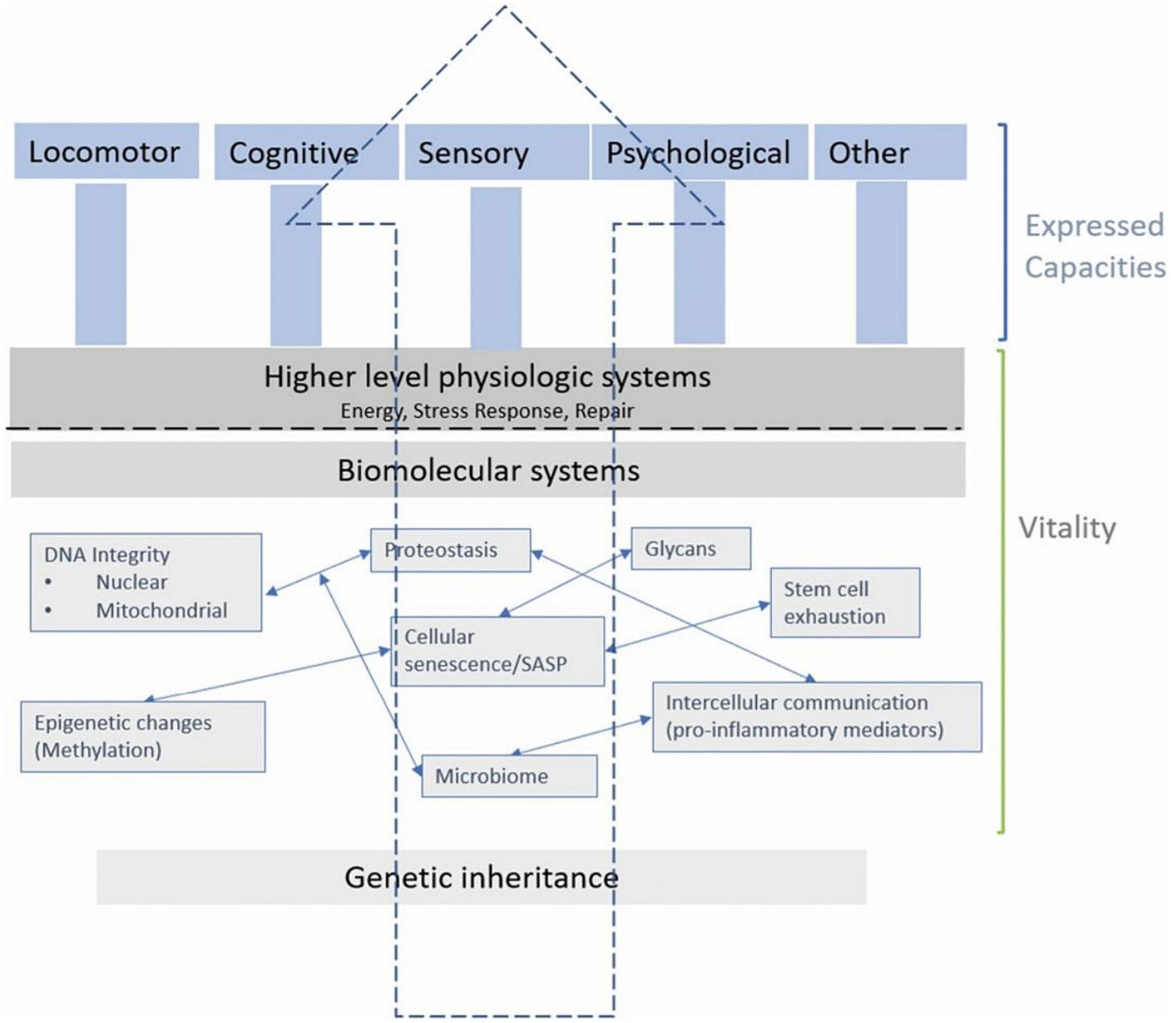
Reproduced with permission from Beard et al. (18)—a conceptual framework explaining vitality and intrinsic capacity.

The concept of intrinsic capacity, introduced by Beard et al. (34) refers to individuals’ physical and mental abilities that enable them to perform daily activities effectively and maintain independence as they age (6, 35). This notion is pivotal in understanding aging, as it shifts the focus from the mere absence of disease to the positive aspects of aging, emphasizing the potential for well-being and functionality into older age. Intrinsic capacity encompasses five domains, including cognitive function, sensory capacity, locomotor function, psychological well-being, and vitality (6). Vitality is described by physical and mental vigor, reflecting an individual’s overall sense of well-being, engagement in life and resilience to challenges associated with aging (7). The longitudinal analysis and validation of intrinsic capacity suggest that it is a robust outcome and applicable across culturally diverse population groups (18, 34). Understanding the complex interplay between nutrition and aging requires a nuanced appreciation of intrinsic capacity and vitality. By nurturing vitality and supporting the various domains of intrinsic capacity, nutrition emerges as a key modulator of aging, offering promising pathways for enhancing quality of life and functional independence among older adults.


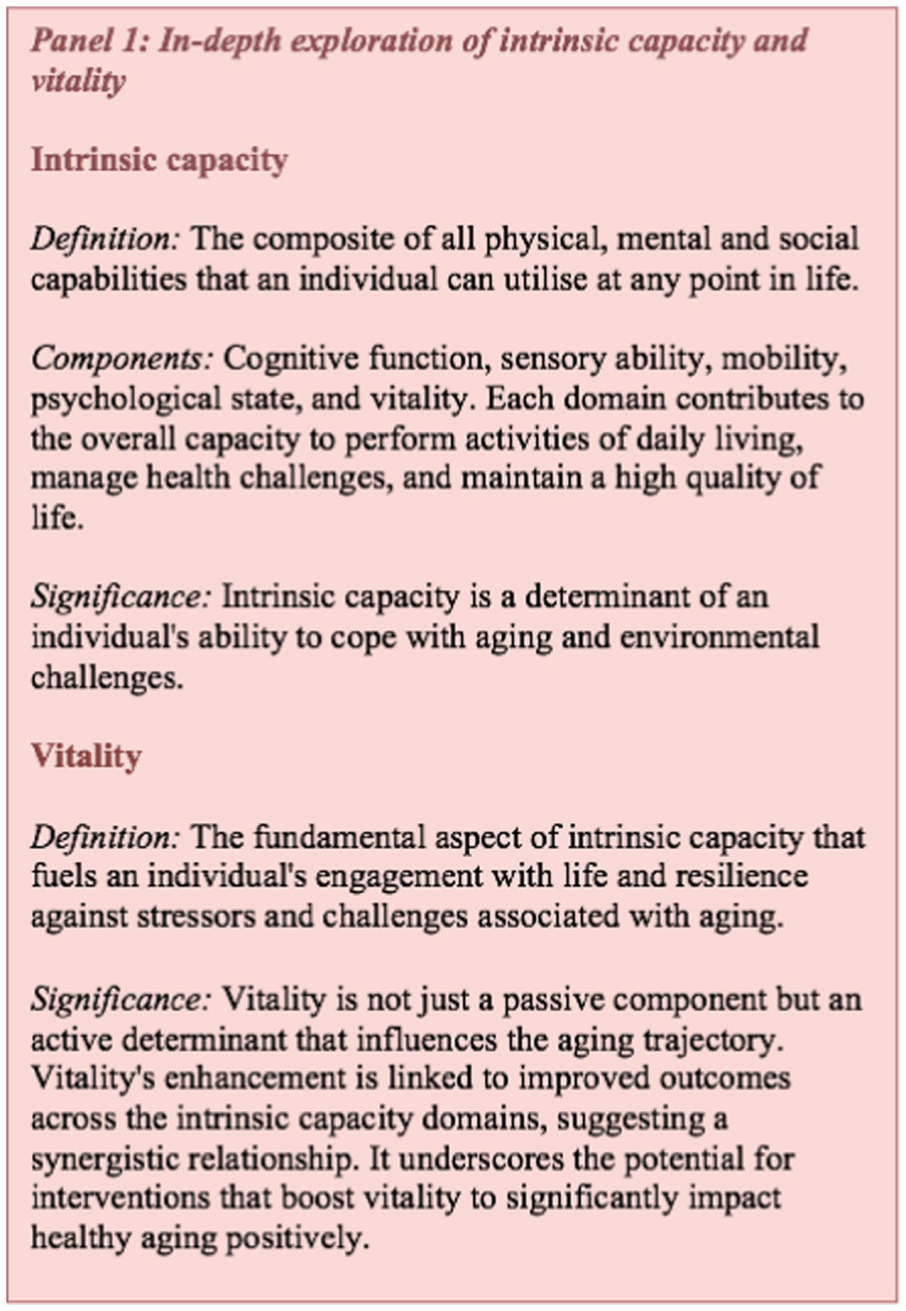
Beard’s framework (Figure 3) is useful for this narrative review since it encapsulates the relevant domains of the cells, organs and potential functional outcomes on which nutrients and phytonutrients might act. Hence, the framework will help to contextualize the literature and elucidate findings. Furthermore, narrative reviews have been criticized for not using existing theoretical or conceptual thinking in their framing, discussion, and consideration of the literature (36).

### 1.3 A framework to conceptualize vitality, intrinsic capacity, and expressed capacities in aging

Enhancing the intrinsic capacity of individuals early in life is likely to have a substantial impact on maintaining independence in older age, thereby reducing the impending strain on healthcare and social support systems globally. Implementing policies and healthcare strategies focused on healthy aging is becoming increasingly crucial as we face an unprecedented global aging population.

Targeting cellular mechanisms involved in the hallmarks of aging can induce shifts in biomolecular systems and organs, affecting vitality (determined by genetic inheritance and other environmental factors) (19) which in turn can modulate intrinsic capacity and expressed capacities (measured as, e.g., locomotor, psychological, cardiorespiratory abilities and more). A recent consensus by a WHO working group underscores the role of vitality as a foundational element of intrinsic capacity (7). While frailty might be considered as an “accumulation of deficits,” the notion of vitality advances thinking in this area toward considering the trajectories of aging.

The overall architecture of this conceptual framework demonstrates the complex interplay between biological factors at distinct levels that cumulatively affect vitality, illustrating how biomolecular and physiological systems interconnect to shape the health and functionality of an organism.

### 1.4 Phytonutrients in the context of aging, vitality, and intrinsic capacity

The relevance of plant-based diets for aging may be attributed, in part, to their high content of nutrients and phytonutrients. Several studies suggest that phytonutrients are an important dietary constituent for healthy aging as they modulate oxidative stress, inflammation, and various signaling pathways involved in the hallmarks of aging, potentially playing a key role in preventing age-related diseases, maintaining normal metabolism, and supporting overall quality of life (37–46).

The “hallmarks of aging” were initially described by López-Otín et al. (20) and further revised a decade later (47). The 12 hallmarks have been extensively described in the literature as being genomic instability; telomere attrition; epigenetic alterations; loss of proteostasis; deregulated nutrient sensing; cellular senescence; mitochondrial dysfunction; stem cell exhaustion; altered intercellular communication; disabled macroautophagy; chronic inflammation and age-related dysbiosis. Although these hallmarks are independent drivers of aging, they are highly interconnected as they share common pathways, namely the sirtuins, mammalian target of rapamycin (mTOR), the nuclear factor erythroid 2–related factor 2 (Nrf2), and the nuclear factor kappa (NF-kb) signaling pathways (20, 47).

Plant-based solutions that attenuate cellular hallmarks of aging may confer benefits to subsequent tissues, organs, and systems (48), and potentially contribute to the intrinsic capacity of aging adults and therefore have the theoretical potential to influence the healthspan of this population (18). For instance, consumption of flavonoids, commonly found in fruits, vegetables, cocoa, wine, and tea, have demonstrated significant efficacy in reducing cellular damage through regulating the phosphoinositide 3-kinases (PI3K)/Akt/mTOR pathway, activation of sirtuin 1 (SIRT1), forkhead box, subgroup O (FOXO) and suppressing pro-inflammatory pathways in aged animal models (49). Inflammation being an important hallmark of aging, the observed anti-aging effect of flavonoids is likely to be mediated by their modulation of molecular pathways driving this hallmark.

However, the scientific literature on this topic presents a varied range of findings. A comprehensive analysis of the current evidence is necessary to discern the effects of different phytonutrients on the key aspects of aging and the development of age-associated conditions. This would help in identifying plant-derived compounds that have potential benefits for human aging. Moreover, existing reviews have not yet aligned their findings with a theoretical model of healthy aging that emphasizes vitality and intrinsic capacity.

### 1.5 The modulatory effect of nutrition on the hallmarks of aging

In the exploration of the molecular mechanisms of how nutrition regulates the aging process, recent advancements in “nutrigerontology” have highlighted the potential of dietary patterns and interventions to modulate the hallmarks of aging, aiming to extend healthspan and combat age-related diseases (33). López-Otín et al. emphasize the crucial role of dietary modulation in addressing the hallmarks of aging, suggesting that it is a viable approach to slow down the degenerative processes (47).

Key findings reveal that caloric restriction, specific nutrient supplementation, and adherence to diets rich in defined nutrients and bioactive compounds, such as NAD+ precursors, omega-3 fatty acids, and polyphenols can influence critical cellular pathways—ranging from mitochondrial function and telomere maintenance to intercellular communication and chronic inflammation (19). Among these bioactive compounds, polyphenols like resveratrol, quercetin, and epigallocatechin gallate have garnered attention for their ability to modulate nutrient sensing pathways including AMPK, SIRTs, and mTOR, which are pivotal in enhancing cellular energy efficiency, orchestrating appropriate metabolic responses, and promoting longevity while reducing disease risk (50, 51). Their impact extends to improving metabolic health and stress resistance through the activation of AMPK and SIRT1, and potentially decreasing cancer risk by inhibiting the mTOR pathway, thus supporting vital cellular processes like autophagy and mitophagy. Additionally, by modulating nutrient sensing pathways, polyphenols exhibit senolytic properties, allowing them to selectively induce the death of senescent cells—cells that have stopped dividing and thus contribute to aging and various chronic diseases (52, 53). Polyphenols, therefore, may have the potential to help improve tissue function and reduce inflammation associated with aging.

Furthermore, polyphenols are recognized for their role in mitigating immunosenescence—a gradual decline in the immune system’s functionality with age—by reducing senescent cell accumulation, decreasing cellular oxidative stress, and modulating immune function through epigenetic mechanisms (54, 55). Their ability to alter the gut microbiome composition, promoting the growth of beneficial bacteria, further supports immune function via the gut-immune system axis (56). These dietary strategies, including the enhancement of microbial diversity that affects systemic health by modulating neuroendocrine and inflammatory pathways, have been shown to regulate cellular mechanisms involved in aging, such as influencing metabolic health and potentially altering the course of aging and disease onset (57). The impact of polyphenols on immunometabolism highlights their potential in integrating metabolic and immune responses to maintain health and delay age-associated diseases (58).

Collectively, these findings underscore the intricate link between diet, cellular health, and healthy aging, offering promising avenues for dietary interventions to improve aging outcomes. While healthy, balanced diets may provide important quantities of phytonutrients from dietary sources (such as anthocyanins from berries, flavanones from citrus fruits and chlorogenic acid from coffee) some dietary patterns contain very low amounts of plant foods (59) and would benefit from dietary modification or supplementation. In the next sections, therefore, we will specifically focus on the potential role of phytonutrients to influence aging processes and relevant outcomes, against the backdrop of Beard et al. framework highlighted in Figure 3.

The relationship between diet and chronic disease is well established. High intakes of fruits and vegetables have been associated with reduced risk of chronic disease and mortality (60–62). The investigation of plant compounds involved in the mitigation of disease in aging has attracted widespread research. Some phytonutrients have been extensively investigated, whereas others that have recently emerged show promise, but well-designed randomized controlled trials (RCTs) are lacking. The next section discuss a range of phytonutrients, from the well-studied to the emerging and innovative, with the potential to support healthy aging. Furthermore, in addition to the dietary sources of phytonutrients, this review considers studies in which the concentrated form of the compound (or “plant nutraceutical) has been studied.

## 2 Amino acids and amines

### 2.1 Spermidine

Spermidine is a naturally occurring polyamine (63) with highest amounts reported in soybeans, peas, and pears (64), as well as in wheat germ, mushrooms, some nuts, and spinach (65). Spermidine is well-absorbed and rapidly distributed to the body’s tissues (66).

In aging, cellular levels of spermidine and ornithine decarboxylase (ODC), the enzyme involved in the synthesis of spermidine, decline with age (67, 68). Spermidine administration significantly extended the lifespan of yeast, flies and worms, and mice (69). *In-vitro* and *in-vivo* data demonstrates the ability of spermidine to impact the cellular hallmarks of aging via enhancing mitochondrial function, inducing cell growth and proliferation along with exerting anti-inflammatory, antioxidant and anti-senescent effects (63). Spermidine has also been shown to enhance autophagy in cellular and animal models of aging (63).

Epidemiological studies have correlated higher dietary spermidine intake with reduced all-cause mortality (70), including reductions in CVD and cancer-specific mortality (71). The first randomized controlled trial (RCT) to investigate the effect of spermidine supplementation on memory performance in older adults was a pilot study published in 2018 which reported a positive effect in adults aged 60–80 years (72). A further RCT found a positive effect of 3 months of spermidine supplementation on cognitive performance, using bread rolls containing either 3.3 mg or 1.9 mg of spermidine, in adults aged 60–96 years. In particular, those receiving the higher dose of spermidine showed improvements in cognitive performance among those with mild and moderate dementia (65). In one double-blind, randomized, placebo-controlled trial, 100 older adults (age 60–90 years) with subjective cognitive decline were supplemented with 0.9 mg/day of spermidine or placebo for 12 months (73). Results indicated no difference in memory performance and biomarkers in the spermidine supplemented group versus the placebo, though exploratory analysis showed possible beneficial effect of the intervention on inflammation and verbal memory. Overall, human RCTs show mixed results from spermidine supplementation.

The pre-clinical data and emerging studies indicate a potential for spermidine to impact cellular hallmarks of aging, and functional parameters such as cognitive function. Further clinical trials are required to confirm the preliminary data effect in humans, in particular for cellular health, neuro-protective function, and the potential to contribute to vitality and intrinsic capacity in aging.

### 2.2 Ergothioneine

Ergothioneine (EGT) is a naturally occurring amino acid, with good free-radical scavenging activity (74). The antioxidant activity of EGT makes it a potential candidate to offset the cycle of damage to cells via its anti-inflammatory, anti-neurodegenerative and anti-senescence properties demonstrated in cellular and animal models (75–78). EGT can only be obtained from a limited number of foods such as mushrooms, liver, black beans, garlic and oat bran (79, 80).

Absorption studies support the ability of EGT to be absorbed via the intestine, passed into circulation, and transferred into tissues throughout the body (81, 82). EGT levels decline after 60 years of age and more so in individuals with cognitive impairments (83, 84). On the contrary, a relationship between healthier dietary patterns, lower CVD risk, and higher circulating EGT levels has been observed (85, 86).

Human clinical studies have been conducted to investigate the effect on EGT on postprandial triglyceride response (87), oxidative stress and inflammation (82), and effects on lipid peroxidation, DNA damage, urate oxidation, protein carbonylation and C-reactive protein with limited to no effects observed (88). A single study examined the functional effect of EGT supplementation (combined with other anti-inflammatory nutrients and analgesics) on joint pain and range of motion (ROM) in adults with significant improvements over 6 weeks (88).

Despite a lack of data from human RCTs related to EGT, its role in cellular signaling pathways associated with aging, the protection of mitochondria and anti-inflammatory, anti-senescent effects make it a promising candidate for improving vitality and intrinsic capacity (34, 89).

## 3 Polyphenols

Polyphenols are natural compounds synthesized by plants and present in a variety of plant foods. They can be classified as phenolic acids, flavonoids (the largest subclass of polyphenols), polyphenolic amide, and other non-flavonoids (90). Epidemiological data highlights the association between polyphenol and flavonoid intake and a reduction of mortality from all causes and from CVD (91), cancer, diabetes, neurodegenerative disease, and osteoporosis (92). Polyphenols have shown multifaceted effects contributing to healthy aging by attenuating the development and accumulation of senescent cells, cellular oxidative stress, and immune dysfunctions (57); promoting cardiovascular health (93), reducing oxidative stress and inflammation; preventing neurodegenerative diseases, modulating autophagy and gut microbiota (94), and potentially extending healthspan by targeting various cellular mechanisms and signaling pathways.

This section will consider some well-known polyphenols and flavonoids, along with lesser-known emerging candidates, and discuss their potential to influence cellular exposure to oxidative stress, inflammation and a reduced activation of protective stress response pathways related to aging (95).

### 3.1 Flavonoids

#### 3.1.1 Cocoa flavanols

Research into the flavanols from cocoa beans has shown that they possess properties which may offset the risk of chronic disease in aging, in particular CVD and type 2 diabetes (T2D), via their antioxidant and anti-inflammatory potential (96, 97). The main flavanols found in cocoa beans are epicatechin, catechin, and procyanidins, which are associated with various health benefits (98). The absorption and bioavailability of the flavanol compounds have been extensively described elsewhere and are well-understood, in particular for epicatechin (99). However, there is less information in relation to the complex metabolism of flavonoids by gut microbial pathways (100). It is thought that the active component of polyphenols might not be the polyphenols themselves, but their active metabolites (101).

The antioxidant potential of cocoa flavanols were first described by Waterhouse et al. who reported that cocoa polyphenols were superior to red wine polyphenols in their ability to inhibit LDL oxidation (102). Cell models have since elucidated that cocoa polyphenols are likely to prevent oxidation by acting on tumor necrosis factor alpha (TNFα), enzymes cyclo-oxygenase-2 (COX-2), C-reactive protein and soluble adhesion molecules (101) to down-regulate inflammation. However, some authors do not agree with the notion that flavonoids act as antioxidant molecules, but rather modulate the antioxidant response by targeting cellular pathways (103).

Several human intervention studies have demonstrated that cocoa and cocoa-containing products are associated with reducing CVD risk (104) via increasing HDL-cholesterol (105), improving endothelial function (106, 107), reducing blood pressure (108), decreasing blood triglycerides (104, 105), and reducing markers of oxidative stress (109). A large clinical trial in the US (COSMOS) reported that while cocoa extract supplementation did not reduce CVD events, it reduced deaths from CVD by 27% (110). Cocoa has also been found to improve insulin sensitivity in human RCTs (107, 111, 112).

Several studies have examined the effect of cocoa flavanols on cognitive performance in both old and younger populations (113) and have shown to have a potential effect on cognitive performance in aged populations since cocoa flavanols enhance cerebral blood flow in older subjects (114). In older adults (115) and adults with mild cognitive impairment (116), cocoa flavanols have been shown to improve cognitive performance and flexibility, processing speed (117), and working memory (118). However, one study in older adults showed no benefit of flavanols from chocolate, or a cocoa beverage, on cognitive performance (118). Inconsistencies in clinical studies on cocoa flavanols may be due to the variability of cocoa flavanol-containing food matrices along with inter and intra-individual variation in the response to cocoa polyphenols (39, 101). Moreover, cocoa flavanol supplementation was shown to positively affects facial wrinkles and elasticity in moderately photo-aged women (119). Nevertheless, future research should elucidate how the timing, form, and dosage of cocoa flavanols could elicit beneficial neuromodulatory effects in aging populations along with genetic factors that may impact an individual’s response to coco flavanols.

Overall, these studies suggest that flavanols in cocoa beans have multiple beneficial effects on health in aging, including improvements in oxidative stress, inflammation, cardiometabolic health, physical performance, skin health, cognitive function, neuroprotection, vascular function, and may reduce the risk of chronic diseases. While research on the specific effects of flavanols in cocoa beans on aging is ongoing, accumulating evidence suggests that incorporating cocoa or cocoa-derived products rich in flavanols into the diet may have beneficial effects on overall vitality and intrinsic capacity in aging.

#### 3.1.2 Luteolin

Luteolin, a flavone metabolite, and luteolin-7-O-glucoside (LUT-7G) is a dietary derived compound found in carrots, peppers, celery, olive oil, rosemary, artichoke, pomegranate and plant extracts such as chrysanthemum (120, 121). Luteolin and LUT-7G have been shown to have potential anti-inflammatory, antioxidant, neuroprotective, DNA-protective, anti-cancer, anti-diabetic properties along with an ability to modulate cellular signaling pathways in *in-vitro* and *in-vivo* studies (122–130). Luteolin has also been shown to protect heart tissue in a diabetic mouse model via modulating Nrf2-mediated resistance to oxidative stress and NF-κB-induced inflammatory responses (131). Such data places luteolin as an emerging candidate to attenuate cellular hallmarks of aging. In particular luteolin has been cited as having widespread neuroprotective effects in both *in-vitro* and *in-vivo* models of Alzheimer’s disease (AD), Parkinsons disease (PD) and cognitive decline, and is able to suppress inflammation in brain tissue (127, 132).

Epidemiological evidence suggests that luteolin possesses anti-inflammatory and cardioprotective effects. Studies have shown a significant reduction in CVD mortality and decreased incidence of epithelial ovarian cancer associated with higher luteolin intake (133, 134).

Human clinical trials investigating luteolin supplementation are limited, with mixed results. One study examining luteolin’s role in Gulf War Illness, characterized by high levels of inflammatory markers, showed no significant improvement in symptom severity (135, 136). However, in a placebo-controlled trial targeting pre-obese individuals, a phytonutrient blend containing luteolin demonstrated promising cardiometabolic outcomes, including weight reduction and improvements in glycemic and lipid parameters. Authors postulated that the findings may, in part, be due to the ability of luteolin to attenuate adipose tissue inflammation and insulin resistance, as demonstrated in animal models (137).

Overall, more research is required to understand effective human doses, metabolism and absorption of luteolin in order to perform well-designed human clinical trials involving luteolin and LUT-7G (138). Nonetheless*, in-vitro* and *in-vivo* data indicate the potential for luteolin to impact vitality via its neuroprotective, cardioprotective, anti-inflammatory and anti-obesity properties. Further research would help to elucidate any eventual impact of luteolin on vitality and intrinsic capacity in aging.

#### 3.1.3 Fisetin

Fisetin is a flavonoid present in fruits such as strawberries, apples, kiwi and mangoes (139). Fisetin demonstrated pre-clinical potential to offset inflammatory pathways that lead to chronic diseases in aging (140). Chronic inflammation, if left uninhibited, may trigger cellular pathways that lead to CVD, osteoporosis, cancer and neurodegenerative disease, via its inter-connectedness with many of the cellular hallmarks of aging (141). Furthermore, fisetin has been widely studied for its antioxidant effects, along with its ability to inhibit cellular senescence (142). Notably, in a pre-clinical trial, out of 10 flavonoids studied, fisetin was found to be the most potent senolytic, reducing senescence markers in multiple tissues (53). Neurodegenerative diseases linked to mitochondrial dysfunction and ROS may benefit from intervention with fisetin since it has been shown to interact with diverse REDOX signaling pathways, restore mitochondrial function, and contribute to prevention of neuronal cell death (143). Despite encouraging *in-vitro and in-vivo* data on the multiple beneficial effects of fisetin in relation to the hallmarks of aging, its translation to human RCTs has been limited for the time being. Small scale human clinical trials focus on fisitin supplementation post cardiovascular events and inflammatory conditions (such as Gulf War Illness) with mixed effects (144–148). Its poor absorption in humans has limited its therapeutic potential. To address this issue, an innovative study proposed encapsulating fisetin into a fisetin-loaded dietary fiber hydrogel scaffolds to improve its delivery and bioavailability in human subjects (149).

While further research is needed to fully elucidate the therapeutic potential of fisetin in humans, both pre-clinical and small-scale clinical studies suggest that fisetin holds promise as a natural compound for promoting vitality in aging. Its multifaceted mechanisms of action make it a promising candidate to impact multiple hallmarks of aging, including cellular senescence, mitochondrial dysfunction, and chronic inflammation.

#### 3.1.4 Quercetin

Quercetin is one of the most common and studied flavonoids, present in many different fruits and vegetables such as onions, apples, berries, kale, leeks, asparagus and capers (150). However, dietary intake of quercetin varies depending on the fruit and vegetables intake (151).

It has been shown to have anti-inflammatory, antioxidant, anti-cancer, anti-hypertensive, anti-diabetic, anti-neurodegenerative and cardio-protective properties in pre-clinical models and in human RCTs (144, 152). Several studies have found beneficial effects of quercetin with respect to antioxidant biomarkers and the inhibition of LDL oxidation (145, 153–155). However, a systematic review of RCTs concluded that quercetin supplementation did not lead to a clinically significant effect on plasma lipids, but a dose of >50 mg/day may have a beneficial effect on reduction of triglycerides (156). One study found that quercetin supplementation inhibited platelet aggregation (157) improved some biomarkers of endothelial dysfunction (158) reduced systolic blood pressure (155) and reduced blood pressure in hypertensive patients (159, 160). Furthermore, a systematic review of RCTs concluded that quercetin supplementation of >500 mg/day could significantly reduce blood pressure (161). Together, these studies suggest that quercetin may confer some cardioprotective effects. However, more studies of longer duration are required, in different populations, to confirm these findings and to gain more insight into potential mechanisms of action. A systematic review of *in-vitro* and *in-vivo* data support the potential anti-diabetic effect of quercetin (162). It has been shown to act on signaling pathways such as TNFα, NFkB, AMPK, Akt, and Nrf2 which are implicated in the pathogenesis of T2D and insulin resistance (163). The oral administration of 250 mg/day for 8 weeks improved the antioxidant status among subjects with T2D (164).

Furthermore, quercetin interacts with SIRT1, a key enzyme in cellular processes linked to aging (165, 166). Animal studies suggest increased SIRT1 expression may protect against AD (167), while decreased expression is observed in aging mice (168). Quercetin regulates SIRT1 pathways, potentially initiating protective mechanisms against AD. A randomized trial in older adults showed quercetin supplementation improved reaction time and preserved cerebral blood flow over 40 weeks (169). Further research is needed, but quercetin shows promise for mitigating age-related cognitive decline and supporting neurocognitive health.

Further investigation is warranted to fully comprehend the potential cardioprotective and cognitive benefits of quercetin, a naturally occurring senolytic compound. Its interaction with SIRT1 makes it a promising candidate for targeting cellular senescence and enhancing vitality. However, a deeper understanding of its mechanism of action within specific disease phenotypes is essential for guiding future research and potential applications of quercetin in alleviating chronic conditions associated with aging. Nonetheless, encouraging diets that contain food sources of quercetin, or the inclusion of bioavailable sources of quercetin as supplements may contribute to vitality and intrinsic capacity in aging.

### 3.2 Stilbenes

#### 3.2.1 Resveratrol

Resveratrol is a polyphenol present in plant foods, with top sources being grape skin, red wine, blueberries, and peanuts (44). The polyphenolic nature of resveratrol makes it a powerful antioxidant shown to upregulate the expression of antioxidant enzymes and to reduce mitochondrial superoxide generation by stimulating mitochondrial biogenesis in pre-clinical studies (170). Several studies suggest that resveratrol has the potential to extend lifespan and improve healthspan in various species, particularly through mechanisms involving metabolic regulation, stress resistance, and activation of longevity genes, although its effects may vary across different organisms (171–173). Resveratrol also has demonstrated significant potential to activate autophagy, increase mitochondrial biogenesis, support free radical quenching, and induce anti-inflammatory effects (44). This may occur through stimulating SIRT1 and NRF2 pathways, and by downregulating NF-kB and Akt/mTOR pathways. Consequently, by regulating multiple longevity-related signaling pathways, resveratrol demonstrates considerable potential in addressing various hallmarks of aging, such as low-grade inflammation, compromised autophagy, and deregulated nutrient sensing (44).

Multiple rodent studies have provided evidence supporting the neuroprotective effects of resveratrol. These studies have demonstrated that resveratrol supplementation improves antioxidant status in the brain, reduces concentrations of inflammatory markers, and enhances memory performance in animal models of memory impairment, including working memory, spatial memory, and learning memory (146–148). Specifically, resveratrol has been found to stimulate brain SIRT1 activity while suppressing NF-kb and enhancing AMPK. This activation of AMPK is crucial in protecting against the accumulation of Amyloid-β through the regulation of neuro-inflammation and oxidative stress (174–176).

One clinical trial has demonstrated that resveratrol supplementation for 30 days induces metabolic changes in obese humans, mimicking the positive effects of calorie restriction (177). Resveratrol supplementation significantly improved glucose control and insulin sensitivity in persons with T2D but did not affect glycemic measures in nondiabetic persons (178). Overall, resveratrol is regarded as a caloric restriction mimetic (CRM) based on evidence from clinical trials demonstrating its ability to replicate some of the metabolic benefits observed with caloric restriction.

Resveratrol has popularly been investigated for its cardioprotective benefits and has been studied in models of CVD (148). In humans, clinical trials have shown contradicting results in terms of the cardioprotective effect of resveratrol. In fact, while consistent reductions in inflammatory markers and improvements in endothelial function are observed, the hypolipidemic, hypoglycemic, and hypotensive effects of resveratrol remain inconclusive (179). Regarding neuroprotection, administering resveratrol orally led to dose-dependent increases in cerebral blood flow during task performance. However, cognitive function remained unaffected in one RCT (179).

Resveratrol is rapidly metabolized after absorption, leading to low bioavailability in its unmetabolized form (180). This may be one of the reasons behind the variability seen in clinical trials. For instance, in a study investigating the effect of resveratrol on blood pressure, only doses higher than 300 mg/day were shown to reduce systolic and diastolic blood pressure, possibly compensating for the low bioavailability of resveratrol (181). Indeed, its bioavailability was shown to depend on the dose administered, and other bioactives contained in the meal (182). Co-ingestion of piperine and quercetin with resveratrol increased its bioavailability in animal studies (182) however further human studies are required to confirm this outcome.

In summary, resveratrol has demonstrated significant benefits as an antioxidant and anti-inflammatory bioactive in model organisms and humans, making it a potential positive contributor to vitality. While resveratrol shows promise as a metabolic enhancer and caloric restriction mimetic, clinical trials have yielded mixed results, particularly concerning its cardioprotective effects. Further research is necessary to clarify its therapeutic potential and optimize its efficacy in promoting vitality and health in aging.

#### 3.2.2 Pterostilbene

Pterostilbene is a naturally occurring polyphenol most abundantly found in blueberries (183), with lower concentrations in other plant sources such as grapes, cranberries, almonds, and peanuts (184). Yet, the quantity of pterostilbene in blueberries may be too low to induce health benefits which means that delivery of pterostilbene via dietary supplements would enable provision of nutritionally relevant amounts (185).

Pterostilbene is a 3,5-dimethoxyl-resveratrol, which has been reported to have superior bioavailability according to data in animal models (186). Many *in-vitro* and *in-vivo* studies have demonstrated the potential vitality enhancing effects of pterostilbene via its ability to exert anti-inflammatory, antioxidant, neuroprotective (187, 188) and cardioprotective effects (189–191). It is understood to elicit these beneficial effects via its action on mitochondrial oxidative stress, mitochondrial biogenesis and mitochondrial apoptosis (192). In addition, there is evidence pterostilbene may have the potential to decrease glucose and increase plasma insulin levels in animal models of T2D (193, 194).

Unfortunately, human RCTs using pterostilbene are scarce. One such study found a beneficial effect of pterostilbene supplementation on systolic and diastolic blood pressure (195). However, an interesting observation from the study was that while pterostilbene supplementation improved blood pressure, there was an increase in LDL cholesterol levels. Remarkably, this rise in LDL cholesterol was not observed in participants who received a combination of grape extract and pterostilbene. Grape extract is known to contain various bioactive compounds with potential cardiovascular benefits and combining it with pterostilbene seemed to mitigate the adverse effect on LDL cholesterol levels.

Further research is warranted in order to design human RCTs which can shed light on the required dosage, delivery method, disease-specific formulations, and efficacy in the longer term (196). Nonetheless, the pre-clinical data support pterostilbene as a potential phytonutrient which may promote vitality in aging via effects on cellular pathways related to hallmarks of aging.

### 3.3 Beta-diketones

#### 3.3.1 Curcumin

Curcumin (diferuloylmethane) is a polyphenolic compound extracted from the turmeric (*Curcuma longa* L.) root that is widely used in Asia, as a spice, food additive, and herbal remedy (197). In fact, in Ayurveda, turmeric is prescribed to support immunity and treat respiratory disorders such as asthma (197). In the scientific literature, curcumin is mostly known for its anti-inflammatory and antioxidant properties and has demonstrated efficacy on reducing the severity of arthritis symptoms (198, 199) and glucose impairment (200, 201) as reported in meta-analyses of clinical trials. Curcumin can target multiple pathways involved in inflammation, autophagy, and other hallmarks of aging. However, its clinical application is limited by poor bioavailability, attributed to low water solubility, rapid metabolism, and fast systemic elimination. To address these challenges, various strategies have been employed, including nanotechnology-based approaches (such as liposomes, nanoparticles, and solid lipid nanoparticles), the use of adjuvants (notably piperine, which inhibits metabolic degradation), and formulation modifications (like water-soluble derivatives and polymeric micelles) (202).

Curcumin induces cellular stress responses in normal human skin fibroblasts, potentially offering a useful anti-aging approach by enhancing cellular antioxidant defenses (203). Curcumin and its metabolite, tetrahydrocurcumin, increase lifespan in model organisms by regulating oxidative stress responses and age-related genes (204). Animal studies have facilitated the understanding of molecular mechanisms underlying curcumin benefits. Curcumin triggers the Nrf2 pathway which activates antioxidative enzymes, thereby mitigating oxidative stress and facilitating ROS removal from the cells, ultimately leading to lower lipid peroxidation, lower ROS concentration in tissues, and increased antioxidant capacity (197). In humans, supplementation with 600 mg of curcumin per day reduces circulating malondialdehyde, a marker of oxidative stress, and increases superoxide dismutase, an antioxidant enzyme, in red blood cells, as reported in a meta-analysis of 8 clinical trials (205). These findings highlight curcumin as a promising candidate for targeting oxidative stress associated with aging and supporting healthy aging. This is particularly true for managing age-related diseases like atherosclerosis and T2D, along with their complications such as retinopathy and nephropathy.

The clinical trial literature on curcumin shows promising efficacy in the prevention of atherosclerosis. Accordingly, two meta-analyses of five (206) and two (207) randomized controlled trials, respectively, reported a significant overall increase in flow-mediated dilation, generally associated with better vascular health, in both healthy and metabolically unhealthy individuals following daily curcumin supplementation. In addition, there is good evidence supporting the role of curcumin in reducing LDL-cholesterol, triglycerides, and increasing HDL-C levels in healthy subjects as well as those at risk of CVD (205, 208, 209).

Although the anti-inflammatory capacity of curcumin may originate from its antioxidant effects, this bioactive has also shown to directly modulate the inflammatory cascade through its inhibitory effect on NF-kB, a known regulator of pro-inflammatory gene expression and promoter of inflammatory cytokines and chemokines (210). Research on curcumin supports its role in attenuating systemic inflammation and reducing symptoms of age-related inflammatory conditions such as osteoarthritis (199, 211), improving CVD risk factors, and reducing severity of non-alcoholic fatty liver disease (212, 213).

Curcumin affects multiple nutrient sensing pathways, including the insulin/insulin growth factor, AKT, and mTOR pathways, crucial for aging hallmarks. *In vitro* studies demonstrate curcumin’s suppression of mTOR and FOXO signaling while enhancing AKT signaling. These effects correlate with lifespan extension in multiple model organisms (197). Curcumin’s signaling effects extend to systemic protection in vital organs like the brain. Animal models of cognitive deficit or neuroinflammation reveal curcumin’s ability to reduce brain tissue inflammation, neuronal loss, and cognitive impairment. However, human studies on curcumin and cognition are limited and yield inconsistent results (214).

In summary, curcumin emerges as a widely utilized phytonutrient with demonstrated antioxidant properties across *in vitro*, animal studies, and human clinical trials. Its potential as an anti-aging bioactive is underscored by its mechanism of action, which involves suppressing mTOR and FOXO pathways while upregulating the P13K–AKT cascade. Notably, curcumin influences several hallmarks of aging, including nutrient sensing, oxidative phosphorylation, and chronic inflammation. Although further research is necessary to validate its role in aging, numerous clinical trials substantiate its efficacy in mitigating inflammatory conditions, endothelial impairment, metabolic deregulation, and CVD—each of which may significantly impact expressed capacities in older adults. As a low-cost intervention, curcumin holds promise for enhancing vitality and intrinsic capacity in aging, thereby warranting continued investigation.

### 3.4 Benzo-coumarins

#### 3.4.1 Urolithin A

Urolithin A (UroA) emerges as a pivotal metabolite derived from the gut microbiota’s conversion of ellagitannins and ellagic acid, abundantly found in red fruits, pomegranates, and nuts. This conversion exhibits considerable variability, influenced by individual age and health status. In a series of *in vitro*, animal, and human studies, a pure form of urolithin A was shown to upregulate genes and activate pathways involved in mitophagy (215), activate antioxidant pathways (216) and reduce oxidative stress markers (217, 218), improve age-related functional health (219, 220), and even increase the lifespan in an aging model (221). In human cell lines, particularly dermal fibroblasts damaged by UV light, UroA has been shown to activate the NRF2 pathway, thereby boosting ROS scavenging and the release of antioxidant enzymes (222). This effect is partly mediated by the SIRT3-FOXO3-PINK1-PARKIN network, which plays a crucial role in mitophagy. Remarkably, treatment with UroA has led to significant reductions in inflammatory cytokines such as TNF-α and IL-6 in studies involving microglia and neural cells (215). Inducing mitophagy has a beneficial effect on mitochondrial health, an effect that has been observed in healthy volunteers after UroA interventions. Significant decreases in acylcarnitines, C-reactive protein, and ceramides—markers of mitochondrial dysfunction and age-related diseases—were observed in middle-aged and older volunteers post-UroA intervention (219, 220).

The functional effects of UroA have been further investigated in the context of age-related diseases. Significant improvements in neuroinflammation and memory impairment have been noted in mice (223), while human studies have reported enhanced muscle strength and exercise performance in middle-aged and older adults (219, 220). Clinical trials focusing on urolithin-producing foods like raspberries, strawberries, and pomegranate have also highlighted potential health benefits. For instance, the consumption of raspberry juice was associated with improved endothelial function, correlating positively with UroA plasma concentration (224). Two clinical trials (225, 226) investigated the consumption of strawberries (226) and pomegranate extract in relation to microbiota compositional changes, reporting an increased abundance of strains associated with longevity (226) and a healthy metabolic profile (225, 226). Although these results are very promising, further studies isolating the effect of UroA itself are warranted to support the emerging evidence on UroA and functional benefits linked to the hallmarks of aging and potential contribution to vitality.

In summary, the mechanistic effects of UroA on mitophagy, mitochondrial health, oxidative balance, and low-grade inflammation could potentially translate into clinically significant advantages for a range of age-related conditions, including sarcopenia, osteoarthritis, Alzheimer’s disease, and more. Clinical evidence supports UroA’s enhancement of muscle strength and metabolic health, with pre-clinical studies suggesting broader anti-aging effects on organs and systems such as the brain and skin.

## 4 Glucosinolates

### 4.1 Glucoraphanin

Glucoraphanin (GPh) is a glucosinolate found in cruciferous vegetables such as broccoli, particularly concentrated in broccoli sprouts, cauliflower, kale, cabbage and Brussel sprouts, and has garnered attention for its antioxidant and anti-inflammatory properties (227–230).

GPh is converted into sulforaphane (SPh) through a process that involves an enzyme known as myrosinase (230). This conversion can occur within the plant itself, as well as through the action of the oral and gut microbiota after ingestion (231). SPh is capable of interacting with many different molecular pathways associated with aging (229). In particular, SPh is a potent inducer of phase 2 enzymes which may help cells to better detoxify and remove potentially harmful compounds (232). There is also a genetic influence which may mediate the benefit of GPh consumption. About 50% of the population possess the glutathione S-transferases 1 genotype (GSTM-1) and may gain additional benefit from GPh ingestion, versus those without the genotype who excrete more SPh in 24 h (233).

Pre-clinical evidence, largely from cell culture and animal studies, has indicated that sulforaphane may have a role in the prevention and amelioration of various conditions such as certain cancers, T2D, and asthma due to its activation of Nrf2 signaling and resulting induction of cytoprotective enzymes (234).

Clinical trials involving broccoli sprout preparations, which contain high levels of GPh, began in the late 1990s (235). These trials have evaluated the safety, acceptability, bioavailability, and pharmacokinetics of these preparations. However, it should be noted that most clinical studies to date have used broccoli sprout preparations rather than pure GPh or SPh, and it remains to be determined whether there is bioequivalence in these interventions. A systematic review cited 8 human RCTs which have examined the effect of broccoli sprouts on various outcomes (236). In a Chinese study, a broccoli sprout containing beverage was consumed for 12 weeks, providing 600 μmol GPh and 40 μmol SPh. The authors reported that SPh was capable of binding and excreting airborne pollutants (237).

Two human clinical trials demonstrated that diets containing 400 g of high-GPh broccoli per week, for 12 weeks, can significantly lower LDL-C levels, versus diets containing 400 g of standard broccoli per week (238). Moreover, among healthy, overweight subjects consuming 30 g of broccoli sprouts per day led to a significant decrease in inflammatory markers as well as in body fat mass (239). A further study demonstrated that a SPh-rich broccoli sprout extract improved liver function by reducing oxidative stress in Japanese men with fatty liver (240). Another small trial randomized 14 patients to a high-glucosinolate diet for 14 days prior to knee replacement surgery. They found that glucosinolates were able to penetrate the knee joint and synovial fluid (241). Such trials pave the way for the exploration of GPh on osteoarthritis of the knee. This builds on prior *in-vitro* and *in-vivo* data demonstrating SPh can protect cartilage destruction (242).

*Helicobacter pylori* (*H. pylori*) infected patients who consumed 70 g/day of broccoli sprouts for 8 weeks displayed decreased markers of *H. pylori* colonization and reduced markers of gastric inflammation (243). A further study found that an aqueous broccoli seed extract reduced levels of pro-inflammatory cytokines and led to greater *H. pylori* eradication versus the placebo (11.1% vs. 3.7% at 60 days) (244). Eradication *of H. pylori* infection is associated with a reduced incidence of stomach cancer (245). These findings from clinical trials are supported by similar results from a study in a female mouse model infected with *H. pylori* (243).

In conclusion, emerging studies highlight the potential health benefits of broccoli, broccoli sprouts, broccoli seeds and sources of myrosinase in the diet, beyond the usual consideration of the nutrient contributions that vegetables make to the diet. Nonetheless, studies are somewhat sparse and heterogeneous. Future research on the benefits of GPh will open up possibilities for a low-cost dietary intervention to enhance antioxidant and anti-inflammatory pathways which may influence vitality capacity in aging.

## 5 Carotenoids

### 5.1 Astaxanthin

Astaxanthin, a xanthophyll carotenoid, has been acclaimed for its anti-aging properties, eclipsing beta-carotene and vastly outperforming vitamins E and C in neutralizing ROS (246, 247). Originating from aquatic sources like crustaceans and salmon, where it imparts a distinct orange-pink-red hue, astaxanthin is integral to their diet for both coloration and health (248). Its bioactivity surpasses that of lutein and zeaxanthin, with promising improvements in inflammation and oxidative markers in preclinical studies (249). Given its foundational role in chronic disease mechanisms, astaxanthin’s research trajectory is directed toward its efficacy as an anti-aging agent (250).

Termed a “mitochondrial antioxidant,” astaxanthin has demonstrated defensive capabilities against ROS in cellular studies, safeguarding cell and mitochondrial membranes (251, 252). Human randomized controlled trials (RCTs) have indicated that astaxanthin supplementation can significantly reduce oxidative stress markers, such as malondialdehyde, and inflammatory markers like IL-6, particularly in T2D patients (253). Furthermore, astaxanthin has been shown to improve skin health, evidenced by enhancements in skin appearance, texture, and UV damage protection (44, 254). Clinical studies have also touched on astaxanthin’s potential neuroprotective effects, with certain studies reporting improved cognitive functions, such as verbal episodic memory, in middle-aged adults following astaxanthin supplementation (255).

Consequently, there is emerging potential for astaxanthin to support healthy aging, as highlighted in studies that demonstrate anti-inflammatory and antioxidant properties, skin- aging benefits and potential protection in the context of neurodegenerative diseases. Astaxanthin warrants further investigation as a potential geroprotector with the potential to contribute to vitality and intrinsic capacity in aging.

## 6 Plant extracts

### 6.1 Rosemary

Rosemary (*Rosmarinus officinalis* L.) contains biologically active phenolic compounds, the principal components being rosmarinic acid (RA), carnosic acid (CA) and carnosol.

RA has been described as having a myriad of healthspan-promoting biological activities *in-vivo* and *in-vitro* (256), such as protecting cell membranes from oxidation (257), promoting antioxidant activity against ROS production and IL-6 release in human keratinocytes (258), along with anti-viral, anti-mutagenic, anti-bacterial, analgesic, and anti-inflammatory activities (259–262). RA has also shown promise in helping to avoid the build-up of amyloid-β aggregation by inhibition of aggregation pathways *in-vivo* (263) and may help to improve motor control and extend lifespan of ALS mouse models (264). How RA exerts these beneficial effects at the level of the cell is not fully understood but experts suggest it may be linked to its ability to act as a regulator of TNF-α-induced NF-κB signaling, which is crucial to the balance between cell life and death (265). Two further active compounds CA and carnosol have been found to have anti-inflammatory, anti-tumor and antioxidant activity *in vitro* and *in vivo* (266–268). In particular, the anti-inflammatory effect of CA on brain microglia (269) and anti-depressant effect in mouse models (270) has led to interest in the use of CA for therapeutic treatment in neurodegenerative diseases.

In human clinical trials, rosemary has frequently been studied for its ability to enhance memory and cognitive function. One clinical trial testing 68 university students found that 500 mg of dried rosemary supplementation (aerial parts) for 1 month significantly improved memory performance and sleep quality, while decreasing anxiety and symptoms of depression (271). One RCT provided RA capsules to patients for 8 weeks as an adjunct therapy for depression. The scores on measures of anxiety and depression reduced significantly in the RA treated group versus the placebo therefore RA may be of use in the reduction of anxiety and depression in depressed patients, alongside anti-depressant medication (272). Another RCT found that short-term low-dose rosemary supplementation (750 mg) induced a statistically significant beneficial effect on speed of memory in older adults (mean age, 75 years old) compared to placebo (273). This study also indicates that exceedingly high RA doses (6,000 mg) lead to impairment in cognitive performance, emphasizing the significance of appropriate dosage in optimizing cognitive benefits. Furthermore, an extract of *Melissa officinalis* (*M. officinalis*), which supplied 500 mg of RA daily, was found to be effective in helping to prevent the worsening of neuropsychiatric symptoms related to AD (274). The same extract was found to be beneficial in preventing cognitive decline in non-hypertensive adults without dementia following supplementation with 500 mg of RA for 96 weeks (275).

Moreover, rosemary has often been studied in combination with other botanical ingredients and therapies. For example, an extract of Lemon balm (*Melissa officinalis*), which supplied 500 mg of RA daily, was found to be effective in helping to prevent the worsening of neuropsychiatric symptoms related to AD (274). The same extract was found to be beneficial in preventing cognitive decline in non-hypertensive adults without dementia following supplementation with 500 mg of RA for 96 weeks (275). One RCT found no effect of a proprietary spearmint extract, high in RA, on executive function in adults (276) whereas a trial in older adults with memory impairment found that supplementation with the spearmint extract (900 mg/day) improved working memory and spatial working memory (277). Improvements in cognitive function were also found in young, healthy adults who consumed the high RA spearmint extract (278). A further trial using the same extract reported an improvement in reactive agility among healthy adults who were supplemented with 900 mg of the extract versus placebo (279). More evidence is provided by one study where a combination of sage, rosemary and lemon balm extract was more effective than a placebo in supporting verbal episodic memory in healthy subjects under 63 years of age (280).

Overall, these studies support the notion that rosemary and its constituents may offer a new avenue in combating age-related cognitive decline. The urgent need for effective treatments in the face of devastating neurodegenerative conditions underscores the critical importance of further research into rosemary extracts. Its potential ability to mitigate cognitive decline and oxidative stress marks a significant breakthrough in the pursuit of safe, cost-effective interventions. Rosemary extracts hold promise in revitalizing cognitive vitality and influencing expressed capacities in aging individuals, paving the way for a future where cognitive problems may find formidable adversaries in nature’s own remedies.

### 6.2 Ginger

Although originating in Southeast Asia, ginger (*Zingiber officinale*) has become a globally consumed spice deeply ingrained in various cultures, cherished for its perceived health benefits and medicinal attributes (281). The gingerols, shogaols, paradols, and zingiberene are the principal bioactive compounds (282, 283). Ginger and its constituents may improve vitality and intrinsic capacity via their ability to modulate cellular pathways related to hallmarks of aging, eliciting anti-inflammatory and antioxidant benefits (284–286).

Preclinical research demonstrates that ginger and its bioactive compounds scavenge free radicals and mitigate oxidative stress (286, 287). Studies in animal models have shown that ginger supplementation enhances antioxidant enzyme activity and reduces oxidative damage to cellular macromolecules, thereby promoting cellular longevity (285, 288). Anti-inflammatory properties are also demonstrated both *in vitro* and *in vivo* indicating that gingerols and related compounds inhibit pro-inflammatory mediators such as NF-κB and cytokines (289–291). By modulating inflammatory pathways, ginger may attenuate age-related inflammation and mitigate associated health risks (289). Moreover, ginger and its bioactive constituents exert neuroprotective effects by enhancing antioxidant defenses, reducing neuroinflammation, and modulating neurotransmitter activity (292, 293). Animal models of AD have demonstrated improvements in cognitive function and decreased neuroinflammation following ginger supplementation, implicating its potential in promoting brain health and longevity (294). Fermented ginger was found to reduce synaptic disorder and neuron cell death (295).

Human trials corroborate these findings (286, 296). A meta-analysis of clinical trials examining the effect of ginger on oxidative stress biomarkers concluded that ginger supplementation decreased biomarkers of oxidative stress such as malondialdehyde, and increased antioxidant enzymes such as GPx (297). Moreover, a comprehensive systematic review of RCTs on ginger and human health concluded that ginger was effective in studies related to digestive function and anti-inflammatory action (298). Commonly recognized for relieving nausea and vomiting, this benefit of ginger is also validated by animal and human trials (299). A single dose of ginger (1.2 g) has been shown to stimulate gastric emptying and stomach contractions (300). One gram of ginger root was able to prevent hyperglycemia-evoked slow-wave gastric dysrhythmias potentially via blunting the production of prostaglandins (301). There are several proposed mechanisms via which ginger may alleviate nausea and vomiting via its anti-inflammatory properties, redox signaling abilities, effect on motility and gastric emptying, and vasopressin release (299). Ginger has been found to promote gastric motility, a beneficial function in healthy individuals (302) and those with dyspepsia (303) and to enhance swallowing in older populations (304).

Ginger’s impact extends to various aging-related health issues, including metabolic disorders, CVD, neurodegenerative conditions, and arthritis. Systematic reviews and meta-analyses of controlled trials show that ginger can significantly modulate metabolic health markers—reducing body weight, waist-to-hip ratio, fasting glucose, insulin resistance index, and elevating HDL-cholesterol (305–309). Cardiovascular health, integral to healthspan and longevity, may also be favorably influenced by ginger. Meta-analyses of RCTs suggest that ginger can lower blood pressure, improve serum lipid profiles, and enhance endothelial function, particularly in individuals with hypertension and dyslipidemia (310). Cognitive health, especially in neurodegenerative diseases, benefits from ginger as well. In fact, findings from an RCT demonstrate that ginger extracts, at doses of 400 or 800 mg/day, could improve cognitive performance in middle-aged women (311).

In the context of arthritis, the anti-inflammatory properties of ginger have shown promise in reducing pain associated with osteoarthritis (OA) and decreasing reliance on pain medication (312, 313). A human trial indicated that 500 mg/day of ginger could lower proinflammatory cytokines after 3 months of supplementation (314). While ginger combined with pain medication was more effective than either alone for OA symptom relief, a separate study found no benefit of ginger powder over placebo for knee OA pain (315, 316). In rheumatoid arthritis (RA), ginger supplementation was observed to potentially influence inflammation and immunity genes, as indicated by changes in disease activity scores and gene expression (317, 318).

Overall, both preclinical and clinical studies provide compelling evidence for the potential health-promoting effects of ginger and its bioactive components, particularly in relation to inflammation, oxidative stress, cardiovascular health, neuroprotection, and metabolic regulation. The evidence points to ginger as a promising natural extract to contribute to vitality, intrinsic capacity and potentially expressed capacities in aging. By targeting underlying mechanisms of aging and age-related diseases, ginger holds promise as a natural intervention for promoting healthspan and longevity in human populations. Further studies which elucidate the effective dosage for different age-related diseases, along with an improved understanding of the pharmacodynamics and pharmacokinetics will accelerate our understanding of gingers’ potential to influence healthspan in aging.

## 7 Discussion

**Figure 4:**
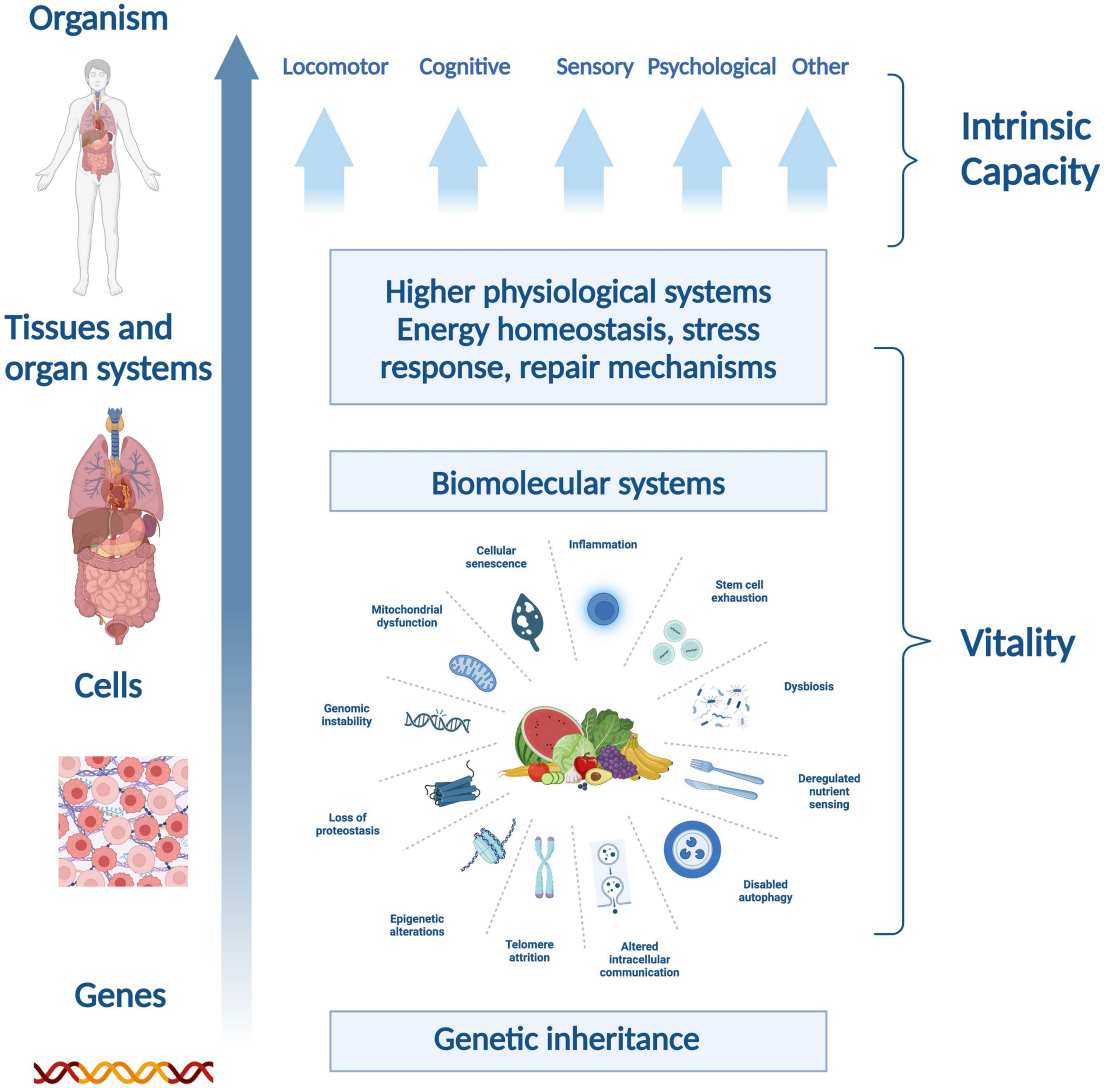
Schematic representation of the relationship between intrinsic capacity and vitality across biological hierarchies.

Our review introduces a novel conceptual thinking in relation to phytonutrients and their ability to impact cellular processes in aging, which may improve vitality and intrinsic capacity (35). Additionally, plants which provide phytonutrients like ginger, curcumin, and rosemary, have been the subject of significant research, could potentially impact the expressed capacities associated with aging. Plant-based diets or supplements that provide phytonutrients could be valuable alongside wider diet and lifestyle interventions in healthy aging (7). The theoretical framework shown in Figure 3 introduces a novel perspective for assessing the efficacy of nutritional components or interventions, proposing a structured framework to support the empirical substantiation of nutrition’s role in preserving and augmenting intrinsic capacity. By scrutinizing the functions of select plant-based nutrients through this lens, we contribute to a refined comprehension of how dietary practices which emphasize the nutrient contribution of plant-foods might be instrumental in advancing healthspan (Figure 4). Indeed, it should be noted that the evidence supporting the efficacy of these phytonutrients spans a spectrum from emerging to multiple human randomized controlled trials, necessitating a balanced and ongoing assessment of nutritional research findings in the context of healthy aging.

Cesari et al. highlights the relationship between nutrition and intrinsic capacity (9). Their research further supports the hypothesis that nutrition significantly influences the core components of intrinsic capacity, thereby affecting vitality and the process of aging. Cesari et al. argue that nutrition is not merely about the prevention of deficiencies but is integral to optimizing health, functionality, and well-being in older adults. A nutritious diet supports all dimensions of an individual’s intrinsic capacity, including cognitive function, physical strength, immune response, and emotional health (6, 9, 25). This conceptual shift underscores the importance of dietary factors in supporting the expressed capacities of aging individuals, and consequently, their functional health (9) (Table 1).

**Table 1:** A qualitative summary of the direction of the evidence in relation to molecular pathways in aging and the potential to influence expressed capacities in aging.

Phytonutrient	Qualitative summary of the direction of the evidence							
	Effect on molecular pathways				Potential effect on expressed capacities			
	No existing human studies	Emerging clinical evidence	Moderate clinical evidence	Good clinical evidence	No existing human studies	Emerging clinical evidence	Moderate clinical evidence	Good clinical evidence
Spermidine			x			x		
Ergothionine				x		x		
Cocoa flavanols			x					x
Luteolin			x			x		
Fisetin			x			x		
Quercetin								
Pterostilbene		x				x		
Resveratrol		x				x		
Curcumin				x			x	
Urolithin A		x					x	
Glucoraphanin			x			x		
Astaxanthin			x				x	
Rosemary			x				x	
Ginger				x				x

Emphasizing a diet rich in plant foods to provide bioactive compounds could become a cornerstone of public health strategies aimed at the aging population (10, 319). Adopting nutritional guidelines that promote such diets has the potential to enhance functional health, decrease comorbidity, and improve overall well-being. Although further clinical research is needed, the use of carefully designed supplements, with adequate safety evaluations, containing standardized, bioavailable extracts may prove helpful in ensuring efficacious doses are consumed. Meanwhile, improving diets by emphasizing plant foods, or Mediterranean-style dietary patterns could lead to reduced healthcare costs and improved quality of life for seniors, aligning healthcare systems and practitioners more closely with the preventive and holistic perspective that the Beard et al. (18, 34) and Bautman et al. (7) advocate.

### 7.1 Crosstalk in molecular mechanisms of action

Integrating the effects of these phytonutrients within the intrinsic capacity framework, it is evident that their roles in healthy aging are interdependent and multifaceted. The cross-over in their mechanisms of action reflects a complex interplay that could potentially support overall vitality. Amino acids such as spermidine and amines like ergothioneine engage in enhancing autophagy and reducing oxidative stress, respectively, sharing common pathways such as the upregulation of sirtuin activity. Polyphenols, including resveratrol, cocoa flavanols, luteolin, fisetin, quercetin, curcumin, and pterostilbene, exhibit overlapping mechanisms like the modulation of the NF-kB pathway, indicating a collective impact on inflammation, cellular senescence, and metabolic regulation (40, 42, 48, 90, 93, 99, 320). The plant extracts from rosemary and glucoraphanin, as well as other nutrients like astaxanthin and urolithin A, contribute to these shared pathways, reinforcing antioxidant defenses, and improving mitochondrial function. The convergence of their effects on molecular targets like mTOR, AMPK, and Nrf2 underscores the potential of a diet rich in these compounds to combat the complex process of aging.

This complex interplay of phytonutrient effects enhances the theoretical framework’s focus on maintaining intrinsic capacity and functional ability, offering a holistic approach to the multifactorial nature of aging. Nutritional interventions that incorporate a range of bioactive compounds could potentially address the interconnected cellular pathways implicated in physiological decline. By doing so, such interventions may serve as a preventive strategy against the cascade of age-related changes. The potential of these compounds to collectively target longevity-regulating pathways suggests that a diet rich in phytonutrients could be a cornerstone for preventive health strategies, aiming to reduce the rate of physiological decline and maintain quality of life in aging populations. Continued research is essential to fully understand the dosage, combinations, and long-term impact of these nutritional interventions.

### 7.2 Reflection on phytonutrients and higher physiological systems

Reflecting on the potential biomarkers of vitality capacity across various physiological systems, such as energy and metabolism, neuromuscular function and immune and stress response, aids in elucidating the potential impact of various bioactive compounds on aging (Figure 4) (7).

Spermidine’s ability to enhance autophagy could theoretically improve metabolic functions and reduce fatigue by clearing damaged proteins and organelles (63). Ergothioneine is recognized for its strong antioxidant properties (76, 89). Thus, higher dietary ergothioneine intake is associated with lower CVD risk in epidemiological studies (86). On the other hand, polyphenols have been largely studied for their antioxidant properties and their effects on vasodilation, energy metabolism and adiposity. (40, 92, 93). GPh reduces LDL cholesterol and improves liver function, indicative of its potential impact on metabolism and antioxidant capacity (229). Astaxanthin can reduce oxidative stress markers and improve lipid profiles, influencing energy metabolism positively (249). UroA may improve mitochondrial health and reduce markers of mitochondrial dysfunction, with associated potential benefits for energy metabolism (218, 219, 223).

In terms of neuromuscular function, polyphenols, especially resveratrol, have been linked to improved muscle strength and function, potentially through their effects on mitochondrial biogenesis and inflammation (92, 101, 144). Moreover, potential benefits for neuroprotection and cognitive health are indicated for resveratrol, cocoa flavanols, curcumin and rosemary extracts, but require further validation in human studies (115, 147, 175, 272). Rosemary’s active compounds as well as curcumin, have demonstrated strong anti-inflammatory effects in some human trials, potentially impacting immune status and stress response (199, 211, 214, 257, 272). There is also moderate evidence for glucoraphanin, astaxanthin and urolithin A to modulate the immune system.

The gaps in understanding the impact of phytonutrients on vitality capacity largely revolve around the complexity of human metabolism and the multifaceted nature of aging. While certain phytonutrients have been linked to improvements in metabolism and inflammation biomarkers the translation to clinically significant outcomes require further investigation. The potential for phytonutrients to impact the attributes of vitality capacity is promising, with several bioactive compounds showing synergistic effects on various physiological systems and some expressed capacities. They may offer non-pharmacological strategies to support healthspan through diet or supplementation. However, realizing this potential requires addressing the aforementioned gaps through well-designed clinical trials, longer study durations, larger and more diverse populations, and standardized outcome measures.

### 7.3 Future directions

Transitioning from the traditional medical paradigm, which primarily focuses on reactive treatment of diseases after symptoms manifest, to a preventive model of care is imperative (15). In this paradigm shift, the emphasis lies on preventive interventions and lifestyle changes to avert future diseases and mitigate physiological decline. Long-term studies are essential to track the aging process accurately; however, they are costly, require sustained funding, and may encounter participant attrition, potentially affecting the consistency of the data. The use of biomarkers could provide more accessible means of monitoring aging, enabling the evaluation of interventions without the need for extensive longitudinal studies (321, 322). Investigating how dietary components like phytonutrients interact with identified biomarkers could advance our understanding of their role in promoting intrinsic capacity.

To effectively preserve intrinsic capacity and vitality in aging, targeting specific biomarkers within panels categorized into physical, cognitive, and metabolic health is crucial. For physical capacity, biomarkers like creatinine and serum cystatin C could be potential candidates as they indicate muscle mass and function (323). Cognitive health might be assessed through brain-derived neurotrophic factor (BDNF) and homocysteine, which are key indicators of neural health and cognitive function (324). Metabolic health is evaluated via insulin sensitivity and lipid profiles, including HDL and LDL cholesterol (325, 326).

Among these, certain biomarkers are particularly likely to be modulated by nutrition: antioxidative markers such as glutathione peroxidase and superoxide dismutase in the physical panel, influenced by dietary antioxidants (327); anti-inflammatory markers like C-reactive protein (CRP) and interleukin-6 (IL-6) within the cognitive realm, modulated by omega-3 fatty acids and polyphenols (328); and Vitamin D levels, vital for both physical and metabolic health, enhanced by dietary intake or supplementation (329). These nutrition-sensitive biomarkers may facilitate a targeted approach to dietary interventions aimed at enhancing overall health and longevity.

Adding a holistic perspective, the systematic review by Leitão et al. (330) addressed how different dietary patterns, particularly those low in carbohydrates and rich in vegetables, fruits, nuts, cereals, fish, and unsaturated fats, not only influence these biomarkers but also play a crucial role in decreasing CVD and obesity risks and protecting brain health (330). This integration underscores the necessity of considering the broad impact of diet when evaluating biomarkers for aging, to formulate nutritional strategies that effectively support preservation of intrinsic capacity and vitality in aging populations. This may pave the way for dietary guidelines and interventions targeting longevity and vitality.

Future studies may also shed light on how these compounds interact at the cellular and molecular levels and improve our understanding of potential combined effects on aging processes. Future research should also explore the dose–response relationships of phytonutrients in clinical settings to optimize dietary recommendations. Exploring the synergy and interaction between different phytonutrients could lead to more effective dietary strategies for aging populations. Identifying which phytonutrients work best together might further enhance their beneficial effects on health.

Therapeutic algorithms may provide a systematic framework for integrating and monitoring the use of phytonutrients alongside established pharmaceutical treatments in managing age-related diseases. Given the complexity of interactions between various compounds and medications, these algorithms can guide healthcare providers in customizing treatment plans that capitalize on the benefits of both natural compounds and conventional medicine while mitigating potential risks. For example, in mice supplementation of 0.05% trans-resveratrol did not interact with warfarin, whereas 0.5% trans-resveratrol enhanced the anticoagulant activity of warfarin (331). Moreover, there is theoretical concern that astaxanthin could interfere with the metabolism of statins prescribed for treatment of cardiovascular diseases. However, other studies have found that astaxanthin could be used as an alternative therapy in statin intolerant patients (332). Through continuous evaluation of potential interactions and effectiveness, therapeutic algorithms could enable healthcare professionals to optimize treatment plans—balancing benefits and risks—thereby enhancing patient outcomes in age-related conditions.

Lastly, personalized nutrition, focusing on tailoring dietary recommendations or nutrient supply based on individual genetic, metabolic, and microbiome profiles, offers a promising avenue for optimizing the efficacy of phytonutrient interventions in healthy aging. This approach could help identify which individuals are most likely to benefit from specific phytonutrients or combinations thereof, based on their unique biological characteristics. By examining genetic markers related to nutrient metabolism and the gut microbiota’s role in phytonutrient bioavailability and effects, personalized nutrition could enhance health outcomes and prevent age-related diseases more effectively.

## 8 Conclusion

In conclusion, our narrative review reinforces the notion that diets, rich in plant foods providing phytonutrients, have the potential to positively influence the aging trajectory. Furthermore, emerging data highlights the potential of phytonutrients as a future intervention to enhance vitality and intrinsic capacity in aging, due to their ability to modulate cellular hallmarks of aging. This highlights the need for a paradigm shift toward preventive nutrition, integrating nutrigerontology into mainstream healthcare practices to support the health and well-being of the aging global population.

## Author contributions

EJ: Conceptualization, Data curation, Formal analysis, Investigation, Methodology, Software, Visualization, Writing – original draft, Writing – review & editing. AK: Conceptualization, Data curation, Methodology, Writing – original draft, Writing – review & editing, Formal analysis. DM: Data curation, Validation, Visualization, Writing – review & editing, Formal analysis. NC: Writing – review & editing. JH: Conceptualization, Writing – review & editing. CH: Conceptualization, Writing – review & editing. MK: Conceptualization, Methodology, Writing – original draft, Writing – review & editing. CL: Writing – review & editing. AR: Conceptualization, Writing – original draft, Writing – review & editing.
